# Nutritional status, dental caries and tooth eruption in children: a longitudinal study in Cambodia, Indonesia and Lao PDR

**DOI:** 10.1186/s12887-018-1277-6

**Published:** 2018-09-14

**Authors:** Jed Dimaisip-Nabuab, Denise Duijster, Habib Benzian, Roswitha Heinrich-Weltzien, Amphayvan Homsavath, Bella Monse, Hak Sithan, Nicole Stauf, Sri Susilawati, Katrin Kromeyer-Hauschild

**Affiliations:** 1 Deutsche Gesellschaft für Internationale Zusammenarbeit (GIZ), L.P. Leviste corner Rufino Street, Makati City, Metro Manila Philippines; 2 Department of Social Dentistry, Academic Centre for Dentistry Amsterdam, Gustav Mahlerlaan 3004, 1081LA Amsterdam, The Netherlands; 3 Department of Epidemiology and Public Health, University College London, Torrington Place 1-19, London, WC1E 6BT UK; 4 Department of Epidemiology and Health Promotion, WHO Collaborating Center for Quality Improvement and Evidence-based Dentistry, College of Dentistry, New York University, 433 First Avenue, New York, NY 10010 USA; 5 Department of Preventive Dentistry and Pediatric Dentistry, University Hospital Jena, Friedrich Schiller University Jena, Bachstraße 18, 07743 Jena, Germany; 6 Faculty of Dentistry, University of Health Sciences Ministry of Health, 7444 Mahosot Rd, Vientiane, Lao People’s Democratic Republic; 7 Department of Preventive Medicine, Ministry of Health, 151-153 Kampuchea Krom Avenue, Phnom Penh, Cambodia; 8 The Health Bureau Ltd., Whiteleaf Business Center, 11 Little Balmer, Buckingham, MK18 1TF UK; 9 Department of Dental Public Health, Faculty of Dentistry, Padjadjaran University, Sekelda Selatan I, Bandung, Indonesia; 10 Institute of Human Genetics, University Hospital Jena, Friedrich Schiller University Jena, Am Klinikum 1, 07740 Jena, Germany; 11 Department of Epidemiology and Biostatistics, College of Public Health, University of the Philippines, 625 Pedro Gil St, Ermita, Manila Philippines

**Keywords:** Dental caries, Tooth eruption, Underweight, Overweight, Growth, Children

## Abstract

**Background:**

Untreated dental caries is reported to affect children’s nutritional status and growth, yet evidence on this relationship is conflicting. The aim of this study was to assess the association between dental caries in both the primary and permanent dentition and nutritional status (including underweight, normal weight, overweight and stunting) in children from Cambodia, Indonesia and Lao PDR over a period of 2 years. A second objective was to assess whether nutritional status affects the eruption of permanent teeth.

**Methods:**

Data were used from the Fit for School - Health Outcome Study: a cohort study with a follow-up period of 2 years, consisting of children from 82 elementary schools in Cambodia, Indonesia and Lao PDR. From each school, a random sample of six to seven-year-old children was selected. Dental caries and odontogenic infections were assessed using the World Health Organization (WHO) criteria and the pufa-index. Weight and height measurements were converted to BMI-for-age and height-for-age *z*-scores and categorized into weight status and stunting following WHO standardised procedures. Cross-sectional and longitudinal associations were analysed using the Kruskal Wallis test, Mann Whitney U-test and multivariate logistic and linear regression.

**Results:**

Data of 1499 children (mean age at baseline = 6.7 years) were analyzed. Levels of dental caries and odontogenic infections in the primary dentition were significantly highest in underweight children, as well as in stunted children, and lowest in overweight children. Dental caries in six to seven-year old children was also significantly associated with increased odds of being underweight and stunted 2 years later. These associations were not consistently found for dental caries and odontogenic infections in the permanent dentition. Underweight and stunting was significantly associated with a lower number of erupted permanent teeth in children at the age of six to seven-years-old and 2 years later.

**Conclusions:**

Underweight and stunted growth are associated with untreated dental caries and a delayed eruption of permanent teeth in children from Cambodia, Indonesia and Lao PDR. Findings suggest that oral health may play an important role in children’s growth and general development.

**Trial registration:**

The study was restrospectively registered with the German Clinical Trials Register, University of Freiburg (trial registration number: DRKS00004485; date of registration: 26th of February, 2013).

## Background

The relationship between children’s oral health and general health has become a research subject of growing interest. Dental caries, the most prevalent childhood disease worldwide, commonly remains untreated [1]. Accumulating evidence indicates that dental caries negatively affects children’s nutritional status and growth [2]. Yet, the nature of this relationship remains controversial, both in terms of the direction and its underlying mechanisms. According to recent systematic reviews, some studies reported an association between dental caries and underweight (low Body Mass Index (BMI)-for-age), stunting (low height-for-age) and failure to thrive, whereas other studies found that dental caries was associated with overweight; or they suggested that there is no relationship [3–5].

Evidence supporting a relationship between dental caries and underweight primarily comes from studies conducted in low- and middle-income countries (LMICs), where severity of dental caries is high [6–9]. Children with high caries levels both in the primary and permanent dentition had significantly lower BMI-for-age, and treatment of severely decayed teeth has been associated with an increased rate of weight gain [2]. Several mechanisms have been postulated to explain this relationship, including the direct effect of dental caries on children’s eating ability and nutritional intake [10], as well as indirect effects of chronic dental inflammation on children’s growth via metabolic and immunological pathways [11]. An opposite theory is that undernutrition (underweight and stunting) could predispose a person to dental caries. Chronic undernutrition has been associated with disturbed dental development, including enamel defects (hypoplasia) and delayed eruption of the primary teeth [12, 13]. However, evidence of the effect of undernutrition on the formation and eruption of permanent teeth is less substantial.

A relationship between dental caries and overweight was more apparent in studies conducted in Europe and the United States [3, 4, 14–16]. Notably, these studies often included samples in which underweight children were underrepresented [3]. In all probability, the mechanisms underlying this relationship follow a different pathway; dental caries and overweight are most likely associated because they have dietary risk factors in common that are both cariogenic and obesogenic, such as a sugar-rich diet [4, 17].

Based on the conflicting findings in the literature, Hooley et al. [3] and Li et al. [5] suggested that dental caries and BMI might be related in a non-linear U-shaped pattern, with caries levels being higher in both children with low and high BMI. There is a lack of studies that have tested this hypothesis, since there are few analyses that covered the full range of anthropometric measurements including underweight, normal weight and overweight (weight status), as well as stunting. In Southeast Asia, dental caries levels are among the highest worldwide, with a prevalence ranging between 79 to 98% in six-year-old children [18, 19]. Undernutrition remains a major public health concern in most countries of the region, yet obesity is also on the rise due to socioeconomic development, globalization and related shifts in dietary intake and physical activity patterns through the nutrition transition [20]. This coexistence of both childhood underweight and overweight, also termed as the ‘double burden of malnutrition’, allows analysis of possible non-linear associations between oral health and nutritional status. Hence, the aim of this study was to assess the relationship between dental caries in both the primary and permanent dentition and nutritional status (as indicated by weight status and stunting) in children from Cambodia, Indonesia and Lao People’s Democratic Republic (Lao PDR), over a period of 2 years. A second objective was to assess whether nutritional status affects the eruption of permanent teeth.

## Methods

### Fit for school – Health outcome study

This study used data from the Fit for School - Health Outcome Study (FIT-HOS), conducted from 2012 to 2014 [21]. The study was originally designed to evaluate the effect of the Fit for School (FIT) programme, which is an integrated Water, Sanitation and Hygiene (WASH) and school health programme to improve child health. It implements evidence-based interventions in public primary schools, including daily group handwashing with soap and toothbrushing with fluoride toothpaste, biannual deworming, and the construction of group washing facilities [22, 23].

The FIT-HOS was a longitudinal cohort study with a follow-up period of 2 years. The cohort consisted of children recruited from 82 public elementary schools - 20 schools in Cambodia, 18 schools in Indonesia, and 44 schools in Lao PDR. Half of the schools in each country (*n* = 41) implemented the FIT programme and the other 41 schools implemented the regular government health education curriculum and biannual deworming as part of the respective national deworming programmes. Per school, a random selection of six to seven-year-old children (6.00 to 7.99 years of age) was drawn from the list of enrolled grade-one students. Baseline data of the children were collected in 2012, and the same children were re-examined 24 months later in 2014. Full details of the study procedures, the selection of schools and the power calculation are described in a previous publication [21]. For the purposes of this study, children were evaluated as one cohort, disregarding the type of school they attended (FIT programme or regular programme).

### Data collection

In each country, a team of local researchers performed data collection on the school ground. For calibration and standardisation purposes, the research teams underwent 3 days of training prior to data collection.

### Clinical dental examination

Clinical dental examinations were performed by four calibrated dentists in the schoolyard or inside a classroom. Dental caries status was scored following the World Health Organization (WHO) Basic Methods for Oral Health Surveys 4th edition [24], using mouth mirrors with illumination (Mirrorlite) and a CPI-ball-end probe. The dt/DT-index was used to score untreated dental caries, by calculating the sum of decayed (d/D) teeth (t/T). The pufa/PUFA-index was used to measure odontogenic infections as a result of untreated dental caries, which scores the presence of teeth with open pulp (p/P), ulceration (u/U), fistula (f/F) and abscesses (a/A) [25]. For both indexes, lowercase letters refer to primary teeth, and uppercase letters refer to permanent teeth. The number of erupted permanent teeth was scored by counting all permanent teeth that had erupted, which was defined as ‘any permanent tooth surface that had pierced the alveolar mucosa’. Kappa-scores for inter-examiner reliability of the dentists ranged from 0.73 to 0.97 (mean *k* = 0.87) for dt/DT and from 0.58 to 1.00 (mean *k* = 0.78) for pufa/PUFA.

### Anthropometric measurement

Two trained nurses obtained children’s weight and height measurements, using standards described by Cogill [26]. Weight was measured to the nearest 0.1 kg using a SECA digital weighing scale. Standing height was measured to the nearest 0.1 cm using a microtoise. The equipment was calibrated at the start of each day and after every 10th child. Children wore light clothes and no shoes during measurement. Measurements were obtained in duplicate, and the average of two measurements was reported. BMI was calculated as weight/height^2^ (kg/m^2^). Weight and height data were subsequently converted to BMI-for-age *z*-scores and height-for-age *z*-scores with the WHO AnthroPlus software, which uses the WHO Growth reference 2007 [27]. *Z*-scores allow comparison of an individual’s weight, height or BMI, adjusted for age and sex relative to a reference population, expressed in standard deviations (SDS) from the reference mean. Cut-offs for BMI-for-age *z*-scores were used to categorize children’s weight status into underweight (< − 2 (SDS), normal weight (≥ -2SDS & ≤ 2SDS) and overweight (> 2SDS). Stunting was defined as a height-for-age *z*-score < -2SDS; scores ≥-2SDs were classified as ‘not stunted’ [28].

### Sociodemographic interview

Sociodemographic information was collected from the children through an interview-administered questionnaire in the respective native language. Demographic information included sex and date of birth, which were cross-checked with the school records. Data on television (TV) ownership, car/motorcycle ownership and number of siblings were collected as proxy indicators of socioeconomic status (SES). These variables have been described as useful proxy measures of SES in LMICs by Howe et al. [29]. Children were asked whether they have a TV at home, and whether they have a car or motorcycle at home, with response options ‘yes’ and ‘no’. The number of siblings was assessed by combining two questions: ‘How many brothers do you have?’ and ‘How many sisters do you have?’

### Data analysis

Data were analyzed using STATA 14 (Stata Corp, College Station, Texas, USA). A *P*-value of ≤0.05 was regarded as significant. Complete case analysis was used to handle missing data. Data were analyzed for each country separately.

The association between dental caries status and odontogenic infections (in further reference: dental caries) and nutritional status was assessed cross-sectionally and longitudinally. First, cross-sectional associations were tested between ^i.^ dt and pufa and nutritional status at baseline at age 6 to 7 years (age 6–7), and ^ii.^ DT and PUFA and nutritional status at follow-up at age 8 to 9 years (age 8–9), using the Kruskall Wallis test for weight status and the Mann Whitney U-test for stunting. Permanent teeth generally start to erupt at the age of 6 years, which means that children’s dentition at baseline mainly consisted of primary teeth, while children’s dentition at follow-up also included permanent teeth. Second, multivariate logistic regression with stepwise backward selection was performed to assess the longitudinal association between dental caries at baseline (dt, DT, pufa and PUFA at age 6–7) and ^i.^ underweight at follow-up (age 8–9) (reference category = no underweight), and ^ii.^ stunting at follow-up (age 8–9) (reference category = not stunted). The regression models were adjusted for sociodemographic factors, number of primary and permanent teeth at baseline and type of school (FIT programme or regular programme).

The association between nutritional status and the number of permanent teeth was assessed cross-sectionally at baseline (age 6–7) and at follow-up (8–9), using the Kruskal Wallis test for weight status and Mann Whitney U-test for stunting. Multivariate linear regression with stepwise backward selection was performed to test the longitudinal association between nutritional status at baseline (age 6–7) and the number of permanent teeth at follow-up (age 8–9). The regression model was adjusted for sociodemographic factors and type of school.

## Results

### Description of the study sample

A total of 1847 children participated in the baseline study – 624 children in Cambodia, 570 in Indonesia and 653 children in Lao PDR. Of those, 76.6% (*n* = 478), 85.3% (*n* = 486) and 81.0% (*n* = 535) were followed-up after 2 years, respectively. Dropout children did not significantly differ from those who were followed-up in terms of their dental caries status and nutritional status at baseline. The mean time interval between baseline and follow-up was 23.88 ± 0.27 months.

The mean age of all children at baseline was 6.7 ± 0.5 years (range 6.0–8.0 years) and 50.2% were boys. The prevalence of underweight and overweight was 7.6% and 7.4% in children at baseline, and 10.2% and 12.3% in children at follow-up, respectively. More than a quarter of children were stunted (30.2% at baseline and 26.2% at follow-up). On average, the number of erupted permanent teeth per child was 5.8 ± 2.8 at baseline and 12.4 ± 3.4 at follow-up. At baseline, the prevalence of dental caries and odontogenic infections in the primary dentition was 94.4% and 69.2%, respectively. Children had a mean dt of 8.4 ± 4.7 and a mean pufa-score of 2.5 ± 2.7. At follow-up, the prevalence of dental caries in the permanent teeth was 41.2% with a mean DT of 0.7 ± 1.2, and the prevalence of odontogenic infections was 7.2% with a mean PUFA of 0.1 ± 0.4. The characteristics of the study samples in the respective countries are described in Table 1.

**Table 1:** Characteristics of the study sample in Cambodia, Indonesia, Lao PDR

	Cambodia		Indonesia		Lao PDR	
	Baseline (*n* = 624)	Follow-up (*n* = 478)	Baseline (*n* = 570)	Follow-up (*n* = 486)	Baseline (*n* = 653)	Follow-up (*n* = 535)
	n (%)	n (%)	n (%)	n (%)	n (%)	n (%)
Gender						
Boys	308 (49.4)	245 (51.3)	295 (51.8)	249 (51.2)	325 (49.8)	272 (50.8)
Girls	316 (50.6)	233 (48.7)	275 (48.3)	237 (48.8)	328 (50.2)	263 (49.2)
Age (years)						
Baseline | Follow-up						
6 to < 7 | 8 to < 9	516 (82.7)	393 (82.2)	388 (68.1)	337 (69.3)	426 (65.2)	358 (66.9)
7 to < 8 | 9 to < 10	108 (17.3)	85 (17.8)	182 (31.9)	149 (30.7)	227 (34.8)	177 (33.1)
Geographical location						
Rural	378 (60.6)	309 (64.6)	–	–	214 (32.8)	187 (35.0)
Urban	246 (39.4)	169 (35.4)	570 (100.0)	486 (100.0)	439 (67.2)	348 (65.1)
Number of siblings^a^						
1 or no siblings	–	144 (30.1)	–	253 (52.3)	–	199 (37.2)
2 siblings	–	137 (28.7)	–	143 (29.6)	–	187 (35.0)
3 or more siblings	–	197 (41.2)	–	88 (18.2)	–	149 (27.9)
TV ownership^a^						
No	–	58 (12.2)	–	3 (0.6)	–	23 (4.3)
Yes	–	418 (87.8)	–	481 (99.4)	–	508 (95.7)
Car / motorcycle^a^ ownership						
No	–	72 (15.1)	–	330 (68.2)	–	41 (7.7)
Yes	–	406 (84.9)	–	154 (31.8)	–	492 (92.3)
Weight status						
Underweight	53 (8.7)	67 (14.3)	45 (7.9)	37 (7.6)	41 (6.4)	46 (8.8)
Normal weight	539 (87.9)	375 (80.1)	443 (78.1)	337 (69.6)	566 (88.2)	434 (82.5)
Overweight	21 (3.4)	26 (5.6)	79 (13.9)	110 (22.7)	35 (5.5)	46 (8.8)
Stunting						
No	410 (66.9)	318 (68.2)	480 (84.8)	401 (83.5)	381 (59.4)	365 (69.5)
Yes	203 (33.1)	148 (31.8)	86 (15.2)	79 (16.5)	261 (40.7)	160 (30.5)
	mean ± sd	mean ± sd	mean ± sd	mean ± sd	mean ± sd	mean ± sd
Number of permanent teeth	5.4 ± 2.7	12.1 ± 3.4	6.0 ± 2.6	12.6 ± 3.0	6.0 ± 3.0	12.6 ± 3.8
dt	9.8 ± 4.5	6.7 ± 3.6	8.2 ± 4.5	5.0 ± 3.4	7.3 ± 4.8	4.4 ± 3.5
DT	0.2 ± 0.6	1.1 ± 1.4	0.1 ± 0.5	0.5 ± 0.9	0.3 ± 0.8	0.6 ± 1.1
pufa	2.6 ± 2.4	2.8 ± 2.1	3.2 ± 3.1	2.7 ± 2.3	1.9 ± 2.4	1.9 ± 1.9
PUFA	0.0 ± 0.1	0.1 ± 0.4	0.0 ± 0.0	0.1 ± 0.4	0.0 ± 0.1	0.1 ± 0.4

^a^Measured at follow-up

Number of missing values at baseline: anthropometric data, *n* = 25; dental data, *n* = 8

Number of missing values at follow-up: anthropometric data, *n* = 21; dental data, *n* = 16

### The association between dental caries and nutritional status

Table 2 shows the cross-sectional associations between dental caries and nutritional status. In Cambodia and Indonesia, dt and pufa were significantly associated with weight status at age 6–7: the mean dt and pufa scores where highest in underweight children and lowest in overweight children. These associations were not observed in Lao PDR. No associations were found between DT or PUFA and weight status at age 8–9, except in Cambodia where the mean DT was again significantly highest in underweight children and lowest in overweight children.

**Table 2:** Dental caries and odontogenic infections according to weight status and stunting in children from Cambodia, Indonesia and Lao PDR at age 6–7 years and at age 8–9 years

	Dental caries (mean ± sd)				Odontogenic infections (mean ± sd)			
	Underweight	Normal weight	Overweight	*P* ^a^	Underweight	Normal weight	Overweight	*P* ^a^
	dt at baseline (age 6–7)				pufa at baseline (age 6–7)			
Cambodia (*n* = 53 | 538 | 21)	11.7 ± 4.7	9.6 ± 4.4	8.6 ± 4.2	0.004	3.1 ± 2.4	2.5 ± 2.4	1.7 ± 2.2	0.033
Indonesia (*n* = 45 | 441 | 79)	9.3 ± 5.0	8.3 ± 4.4	6.3 ± 4.5	< 0.001	3.6 ± 2.9	3.3 ± 3.1	2.5 ± 3.1	0.007
Lao PDR (*n* = 41 | 562 | 35)	8.8 ± 5.2	7.2 ± 4.8	6.5 ± 4.4	0.094	1.8 ± 2.1	1.8 ± 2.3	2.1 ± 3.2	0.997
	DT at follow-up (age 8–9)				PUFA at follow-up (age 8–9)			
Cambodia (*n* = 67 | 372 | 26)	1.4 ± 1.4	1.1 ± 1.3	0.7 ± 1.2	0.030	0.1 ± 0.5	0.1 ± 0.4	0.1 ± 0.3	0.985
Indonesia (*n* = 37 | 335 | 109)	0.9 ± 1.3	0.6 ± 0.9	0.4 ± 0.8	0.176	0.2 ± 0.6	0.1 ± 0.4	0.1 ± 0.3	0.751
Lao PDR (*n* = 45 | 427 | 46)	0.8 ± 1.1	0.6 ± 1.1	0.5 ± 1.0	0.537	0.1 ± 0.3	0.1 ± 0.4	0.1 ± 0.3	0.987
	Not stunted	Stunted		*P* ^b^	Not stunted	Stunted		*P* ^b^
	dt at baseline (age 6–7)				pufa at baseline (age 6–7)			
Cambodia (*n* = 409 | 203)	9.6 ± 4.3	10.2 ± 4.8		0.058	2.5 ± 2.3	2.6 ± 2.5		0.992
Indonesia (*n* = 478 | 86)	7.9 ± 4.4	9.6 ± 4.6		0.002	3.0 ± 3.0	4.1 ± 3.6		0.010
Lao PDR (*n* = 377 | 261)	6.9 ± 4.8	7.8 ± 4.9		0.018	1.8 ± 2.2	1.9 ± 2.5		0.666
	DT at follow-up (age 8–9)				PUFA at follow-up (age 8–9)			
Cambodia (*n* = 316 | 147)	1.1 ± 1.4	1.0 ± 1.3		0.496	0.1 ± 0.4	0.1 ± 0.2		0.316
Indonesia (*n* = 399 | 79)	0.5 ± 0.9	0.7 ± 1.1		0.485	0.1 ± 0.4	0.1 ± 0.5		0.867
Lao PDR (*n* = 357 | 160)	0.7 ± 1.2	0.5 ± 0.9		0.294	0.1 ± 0.5	0.1 ± 0.3		0.820

^a^Kruskall Wallis Test, ^b^ Mann Whitney *U*-Test

In all three countries, a higher mean dt was significantly associated with stunting at age 6–7. In Indonesia, stunted children also had significantly higher levels of pufa at age 6–7, but not in Cambodia and Lao PDR. No significant associations between DT and PUFA and stunting at age 8–9 were found.

Table 3 shows the association between dental caries at age 6–7 and underweight at age 8–9. In Cambodia, higher dt and DT at age 6–7 were significantly associated with increased odds of being underweight at age 8–9, after adjustment for age, sex, the number of permanent teeth and stunting. In Lao PDR the same direction of association was found, but only for dt, while Indonesia showed no association between dt or DT and underweight.

**Table 3:** The association between dental caries and odontogenic infections at age 6–7 years and underweight at age 8–9 years of children in Cambodia, Indonesia and Lao PDR

	Cambodia (*n* = 467^a^)		Indonesia (*n* = 478^a^)		Lao PDR (*n* = 522^a^)	
	*OR (95% CI)*	*P*	*OR (95% CI)*	*P*	*OR (95% CI)*	*P*
Weight status at follow-up (age 8–9): no underweight (reference), underweight						
dt (baseline)	1.09 (1.02; 1.16)	0.010			1.09 (1.02; 1.16)	0.011
DT (baseline)	1.75 (1.15; 2.66)	0.009				
Number of permanent teeth (baseline)	0.84 (0.74; 0.95)	0.007	0.82 (0.70; 0.96)	0.015	0.85 (0.75; 0.96)	0.011
Sex						
Boys	1				1	
Girls	0.27 (0.15; 0.50)	< 0.001			0.08 (0.03; 0.22)	< 0.001
Age (baseline)						
6 < 7 years	1		1			
7 < 8 years	2.29 (1.08; 4.83)	0.030	3.97 (1.84; 8.59)	< 0.001		
Stunting (follow-up)						
No			1			
Yes			2.79 (1.32; 5.89)	0.007		

Logistic regression

Variables in the model: dt at baseline, DT at baseline, pufa at baseline, PUFA at baseline, number of primary teeth at baseline, number of permanent teeth at baseline, sex (boys, girls), age group at baseline (6 to < 7 years, 7 to < 8 years), geographical location (urban, rural), number of siblings (1 or no siblings, 2 siblings, 3 or more siblings), TV ownership (no, yes), car/motorcycle ownership (no, yes), stunting at follow-up (no, yes), FIT programme (no, yes)

‘1’ refers to the reference category: no underweight (BMI: SDS ≥ −2)

^a^Number of children with missing values of variables in the model: Cambodia, *n* = 11, Indonesia, *n* = 8, Lao PDR, *n* = 13

The association between dental caries at age 6–7 years and stunting at age 8–9 years is presented in Table 4. In Indonesia and Lao PDR, a higher dt at age 6–7 was significantly associated with higher odds of being stunted at age 8–9, after adjustment for age, number of permanent teeth, weight status, car/motorcycle ownership and geographical location. The same association was found in Cambodia for DT instead of dt.

**Table 4:** The association between dental caries and odontogenic infections at age 6–7 years and stunting at age 8–9 years of children in Cambodia, Indonesia and Lao PDR

	Cambodia (*n* = 467^a^)		Indonesia (*n* = 478^a^)		Lao PDR (*n* = 521^a^)	
	OR (95% CI)	*P*	OR (95% CI)	*P*	OR (95% CI)	*P*
Stunting at follow-up (age 8–9): not stunted (reference), stunted						
dt (baseline)			1.07 (1.01; 1.13)	0.003	1.05 (1.01; 1.09)	0.025
DT (baseline)	1.67 (1.14; 2.43)	0.008				
Number of permanent teeth (baseline)	0.74 (0.67; 0.82)	< 0.001	0.89 (0.79; 1.00)	0.044	0.82 (0.76; 0.89)	< 0.001
Age (baseline)						
6 < 7 years	1		1		1	
7 < 8 years	3.62 (2.02; 6.51)	< 0.001	3.01 (1.69; 5.37)	< 0.001	2.27 (1.44; 3.57)	< 0.001
Weight status (follow-up)						
Underweight	1		1		1	
Normal weight	0.60 (0.34; 1.06)	0.079	0.44 (0.21; 0.94)	0.034	0.77 (0.40; 1.48)	0.431
Overweight	0.13 (0.03; 0.62)	0.011	0.10 (0.03; 0.33)	< 0.001	0.16 (0.04; 0.59)	0.006
Geographical location						
Rural					1	
Urban					0.56 (0.37; 0.85)	0.006
Car/motorcycle ownership						
No	1					
Yes	0.48 (0.27; 0.87)	0.015				

Logistic regression

Variables in the model: dt at baseline, DT at baseline, pufa at baseline, PUFA at baseline, number of primary teeth at baseline, number of permanent teeth at baseline, sex (boys, girls), age group at baseline (6 to < 7 years, 7 to < 8 years), geographical location (urban, rural), number of siblings (1 or no siblings, 2 siblings, 3 or more siblings), TV ownership (no, yes), car/motorcycle ownership (no, yes), weight status at follow-up (underweight, normal, overweight), FIT programme (no, yes)

‘1’ refers to the reference category: not stunted (Height: SDS ≥ − 2)

^a^Number of children with missing values of variables in the model: Cambodia, *n* = 13, Indonesia, *n* = 8, Lao PDR, *n* = 14

### The association between nutritional status and the number of erupted permanent teeth

The cross-sectional association between nutritional status and the number of erupted permanent teeth is shown in Table 5. In Indonesia and Lao PDR, weight status at age 6–7 and at age 8–9 were significantly associated with the number of erupted permanent teeth: the mean number of erupted permanent teeth was lowest in underweight children and highest in overweight children. In all countries, stunted children had significantly fewer erupted permanent teeth than children with normal height-for-age, both at age 6–7 and age 8–9 (except in Indonesia at age 8–9).

**Table 5:** Number of permanent teeth according to weight status and stunting in children from Cambodia, Indonesia and Lao PDR at age 6–7 years and at age 8–9 years

	Number of permanent teeth (mean ± sd)			
Country (*n* in weight categories)	Underweight	Normal weight	Overweight	*P* ^a^
	Baseline (age 6–7)			
Cambodia (*n* = 53 | 538 | 21)	5.08 ± 2.59	5.38 ± 2.75	5.71 ± 2.81	0.599
Indonesia (*n* = 45 | 441 | 79)	5.53 ± 2.47	5.78 ± 2.48	7.32 ± 2.76	< 0.001
Lao PDR (*n* = 41 | 562 | 35)	5.44 ± 2.67	5.96 ± 2.96	7.49 ± 3.02	0.008
	Follow-up (age 8–9)			
Cambodia (*n* = 67 | 372 | 26)	11.99 ± 3.23	12.09 ± 3.45	12.69 ± 3.96	0.604
Indonesia (*n* = 37 | 335 | 109)	11.97 ± 2.76	12.28 ± 2.87	13.69 ± 3.32	< 0.001
Lao PDR (*n* = 45 | 427 | 46)	11.20 ± 3.70	12.53 ± 3.62	14.67 ± 4.18	< 0.001
	Not stunted	Stunted		*P* ^b^
	Baseline (age 6–7)			
Cambodia (*n* = 409 | 203)	5.84 ± 2.76	4.43 ± 2.44		< 0.001
Indonesia (*n* = 478 | 86)	6.11 ± 2.59	5.20 ± 2.28		0.003
Lao PDR (*n* = 377 | 261)	6.69 ± 2.96	5.03 ± 2.70		< 0.001
	Follow-up (age 8–9)			
Cambodia (*n* = 316 | 147)	12.62 ± 3.38	10.99 ± 3.36		< 0.001
Indonesia (*n* = 399 | 79)	12.68 ± 3.01	12.14 ± 3.09		0.206
Lao PDR (*n* = 357 | 160)	13.14 ± 3.75	11.36 ± 3.42		< 0.001

^a^Kruskall Wallis Test, ^b^ Mann Whitney *U*-Test

Table 6 shows the longitudinal association between nutritional status and the number of erupted permanent teeth. In all three countries, underweight at age 6–7 (except in Cambodia) and stunting at age 6–7 were significantly associated with a lower number of erupted permanent teeth at age 8–9, after adjustment for age, sex, and geographical location.

**Table 6:** The association between weight status and stunting at age 6–7 years and the number of permanent teeth at age 8–9 years of children in Cambodia, Indonesia and Lao PDR

	Cambodia (*n* = 464^a^)		Indonesia (*n* = 480^a^)		Lao PDR (*n* = 516^a^)	
	*ẞ (95% CI)*	*P*	*ẞ (95% CI)*	*P*	*ẞ (95% CI)*	*P*
Number of permanent teeth						
Weight status (baseline)						
Underweight			Reference		Reference	
Normal weight			0.55 (−0.38; 1.48)	0.247	0.52 (− 0.68; 1.73)	0.393
Overweight			1.94 (0.81; 3.07)	0.001	2.98 (1.25; 4.72)	0.001
Stunting (baseline)						
No	Reference		Reference		Reference	
Yes	−1.67 (−2.30; −1.04)	< 0.001	−0.87 (− 1.60; −0.14)	0.019	−2.22 (− 2.83; − 1.62)	< 0.001
Sex						
Boys	Reference		Reference		Reference	
Girls	−1.35 (−1.96; −0.74)	< 0.001	1.07 (0.57; 1.57)	< 0.001	−0.75 (− 1.35; − 0.14)	0.015
Age (baseline)						
6 < 7 years	Reference		Reference		Reference	
7 < 8 years	1.96 (1.13; 2.73)	< 0.001	2.00 (1.45; 2.56)	< 0.001	2.59 (1.97; 3.21)	< 0.001
Geographical location						
Rural	Reference					
Urban	0.77 (0.13; 1.40)	0.018				

Linear regression

Variables in the model: weight status at baseline (underweight, normal weight, overweight), stunting at baseline (no, yes), sex (boys, girls), age group at baseline (6 to < 7 years, 7 to < 8 years), geographical location (urban, rural), number of siblings (1 or no siblings, 2 siblings, 3 or more siblings), TV ownership (no, yes), car/motorcycle ownership (no, yes), FIT programme (no, yes)

^a^Number of children with missing values of variables in the model: Cambodia, *n* = 14, Indonesia, *n* = 6, Lao PDR, *n* = 19

## Discussion

This study investigated the relationship between nutritional status and untreated dental caries, as well as status of eruption of permanent teeth in a community-based sample of children from Cambodia, Indonesia and Lao PRD over a period of 2 years. Findings showed that untreated dental caries in children was significantly associated with underweight and stunted growth. Generally, levels of untreated dental caries in the primary dentition were highest in underweight children, as well as in stunted children, and lowest in overweight children. Untreated dental caries in six to seven-year old children was also significantly associated with increased odds of being underweight and stunted 2 years later. Yet, no consistent associations between dental caries in the permanent dentition and weight status or stunting were found. Hence, the findings of this study did not support the hypothesis of Hooley et al. [3] and Li et al. [5] which suggested that dental caries is associated with both low and high BMI in a U-shaped pattern.

### Discussion of findings related to dental caries and nutritional status

Findings of the current study affirm the results of a number of previous studies, which demonstrated an inverse relationship between dental caries and nutritional status in children [7, 9, 30–33]. These studies have in common that their study population consisted of children with a high caries experience and high caries risk. Most of the studies were conducted in LMICs where dental caries is highly prevalent and commonly untreated, or they included children requiring dental rehabilitation under general anesthesia. This may suggest that the severity of dental caries (the number of caries lesions and caries activity) plays a role in the direction and nature of its relationship with nutritional status. For example, Benzian et al. [8] found that odontogenic infections as a result of untreated decay (pufa/PUFA > 0) was a stronger determinant of low weight in children than dental caries experience (number of decayed, missing and filled teeth (dmft/DMFT > 0)). In the current study, only 1.7% and 6.3% of caries lesions in the primary teeth and permanent teeth respectively were filled or extracted, and most caries lesions concerned decay with advanced progression into the dentine. Therefore, only active caries (dt/DT) was considered in the analysis (rather than dmft/DMFT), which may explain why this study found a stronger association between dt/DT and underweight or stunting in multivariate regression analyses.

There are several explanations of how severe untreated dental caries may be associated with underweight and poor growth in children. Untreated caries can cause pain and discomfort, which negatively affects children’s ability to eat and sleep [9, 17, 34]. Limited ability to eat could lead to poor appetite and reduced nutritional intake, while disturbance of sleep could impair the secretion of growth hormones [35]. Indirectly, chronic inflammation as a result of severe caries with pulpitis could affect growth via immune and metabolic responses. Inflammatory cytokines, for example interleukin-1, can inhibit erythropoiesis, leading to chronic anaemia as a result of suppressed erythrocyte production and haemoglobin levels [36–38]. Inflammation may also contribute to undernutrition through increased metabolic demands and impaired nutrient absorption [11]. Evidence for the mechanisms being causal comes from Acs et al. [39] and the Weight Gain Study [40], which showed a significant increase in weight gain (“catch-up growth”) after extraction of severely decayed teeth in underweight children. However, two randomized-controlled trial in Saudi-Arabia could not confirm these findings [41].

In affluent populations, the relationship between dental caries and nutritional status is likely of a different nature. Studies in industrialized countries have demonstrated positive associations between BMI and dental caries, particularly in the permanent dentition [4, 14–16]. Both diseases have dietary and sociodemographic risk factors in common, which likely underlie the association. As Hooley et al. [3] pointed out, the development of dental caries in affluent populations might be progressing more slowly because of better oral hygiene, higher fluoride exposure and access to oral healthcare. Hence, measurement of dental caries in studies from industrialized countries often included initial enamel lesions or dentine lesions without pulpitis, as well as filled and extracted teeth (rather than untreated caries only), making comparison of results between low, middle and high income countries challenging.

Surprisingly, no significant associations with regards to dental caries in the permanent dentition were found in this study, except in Cambodia. The probable reason for this is that the permanent teeth had just erupted in children at baseline at the age of 6 to 7 years, which means that there was little time in the study for caries to develop in the permanent dentition. The low levels of DT and PUFA at follow-up at the age of 8 to 9 years may have resulted in too little variance to establish significant associations. Previous studies that did find an association between underweight and dental decay in the permanent dentition included children who were at least 3 years older [7, 8, 33]. A probable reason why significant associations could be demonstrated in Cambodia is that the prevalence of dental caries was substantially higher in Cambodia than in Indonesia and Lao PDR. This could potentially be explained by worse general conditions of living and hygiene, which could have affected children’s oral health. Another potential explanation is that the implementation quality of the Fit for School programme (including the toothbrushing activity and exposure to fluoride toothpaste) was poorer in Cambodia as compared to the other two countries.

### Discussion of findings related to nutritional status and the eruption of permanent teeth

The current study also presented evidence for a relationship between nutritional status and the number of erupted permanent teeth. Underweight and stunted children had a delayed eruption of permanent teeth compared to children of normal weight and height, while overweight children showed an accelerated eruption. These findings confirm those of others [13, 42, 43]. Impaired dental development and underweight or stunting likely have common risk factors. For example, nutritional deficiency, including protein-energy malnutrition, may impair dental development via similar mechanisms of influencing skeletal and physical development. Hence, delayed permanent tooth eruption may be one of the manifestations of chronic nutritional deficiencies, making it a valuable indicator of poor overall development in children. The development of permanent teeth follows a sequence over a long period of time, which already starts before or soon after birth. There is evidence that undernutrition during the susceptible stages of tooth development, particularly during a child’s early years, can lead to enamel hypoplasia, making teeth more susceptible to demineralization and dental caries [12, 44]. This suggests that bidirectional effects may exist between undernutrition and dental caries, whereby undernutrition increases the risk of dental caries and vice versa.

### Strengths and limitations

The findings of this study should be interpreted in view of their strengths and limitations. Strengths of the current study were the large community-based sample of children from Cambodia, Indonesia and Lao PDR, the inclusion of both dental caries and odontogenic infections, as well as the full spectrum of anthropometric measurements, and the use of standardized methods to assess oral health and nutritional status by calibrated examiners. Yet, comparison of our results with previous research should be made with caution, since non-uniform parameters have been used in the literature to assess nutritional status, including continuous BMI or BMI *z*-scores or classifications according to WHO references, the 2000 Center for Disease Control and prevention (CDC) growth charts [45] or national references. An important limitation of the study is that no causal inferences are allowed, since the study had only a short follow-up period of 2 years. Furthermore, the study findings are limited to children who attend primary schools. According to data of the World Bank, school enrollment rates of primary school-aged children varied from 92.9 to 97.4% in Cambodia, Indonesia and Lao PDR in 2012 [46]. Hence, a small percentage of children who do not go to school at all could not be represented in the current study sample, yet these children may differ in terms of health and socioeconomic characteristics from those who do attend school.

Data on socioeconomic factors were collected through measurement of TV ownership, car/motorcycle ownership and number of siblings as proxy indicators for SES. Although asset-based measures and family size can be useful proxy indicators for SES in LMICs, they were collected from young children via self-reporting. Possible limitations with regard to the reliability and validity of their response and the socioeconomic data in this study should be kept in mind. Furthermore, this study did not account for a number of other potentially relevant confounders, such as dietary factors, poverty and living conditions. Cambodia, Indonesia and Lao PDR have been experiencing a nutrition transition as a result of economic development and globalization over the last decades [47]. This transition describes a rapid shift in dietary patterns and energy expenditure, which is partially associated with an increased accessibility to nutrient-poor foods that are high in saturated fats and sugars [20]. Particularly the increasing availability and affordability of sugary foods and drinks, also for families from lower SES, pose children at higher risk of developing both dental caries and poor nutritional status. School feeding programmes that provide sugar-rich foods to undernourished children may also contribute to the development of dental caries. To the authors’ knowledge, none of the schools that participated in the study implemented a feeding programme dyring the course of the study, but in nearly all schools children can buy fast food and unhealthy snacks on the school ground. Future studies should include the aforementioned factors, using additional methods of data collection, to explore the potential mechanisms underlying the relationship between oral and nutritional health.

## Conclusions

This study found that untreated dental caries in the primary dentition was associated with underweight and stunted growth in children from Cambodia, Indonesia and Lao PDR. These associations were not found for dental caries in the permanent dentition. The study also provided evidence that underweight and stunting was associated with a delayed eruption of permanent teeth. These findings suggest that oral health may play an important role in children’s growth and general development. Both dental caries and delayed tooth eruption are likely related to chronic rather than acute episodic undernutrition, given the associations found with low BMI-for-age and height-for-age over a period of 2 years.

Findings of this study have important public health implications. In the context of achieving the Sustainable Development Goals [48], in particular goal 2 ‘zero hunger’ to end all forms of malnutrition and goal 3 ‘good health and well-being’, it is of high importance that the underlying determinants of undernutrition and poor development are addressed. Severe dental caries is one of those determinants, which can be effectively tackled through simple, evidence-based and cost-effective measures. These include oral urgent care (often involving tooth extractions) to treat dental infections and address pain and suffering, and promoting the availability and use of fluoride toothpaste to prevent further caries progression and onset of new caries lesions. This should be combined with strategies to reduce the exposure and intake of sugars for effective caries prevention. The Philippines and other contries of the region have already introduced a taxation on sugar-sweetened beverages and regulations on food available in schools [49], which are first steps in the comprehensive prevention and control of non-communicable diseases through upstream policy changes. Promoting good oral health and addressing untreated tooth decay should be among the priority choices in health promotion planning to improve the development and well-being of millions of children that are underweight worldwide.

## Abbreviations

BMI: Body mass index
CDC: Center for disease control and prevention
dmft/DMFT: Number of decayed, missing and filled primary/permanent teeth
dt/DT: Number of decayed primary/permanent teeth
FIT: Fit for School
FIT-HOS: Fit for School – Health Outcome Study
Lao PDR: Lao People’s Democratic Republic
pufa/PUFA: Number of primary/permanent teeth with pulp involvement, ulcerations, fistula and abscesses
SDS: Standard deviations
WASH: Water, Sanitation and Hygiene
WHO: World Health Organization

## References

[1] Kassebaum NJ, Smith AGC, Bernabé E, Fleming TD, Reynolds AE, Vos T, Murray CJL, Marcenes W (2017). Global, regional, and national prevalence, incidence, and disability-adjusted life years for oral conditions for 195 countries, 1990–2015: a systematic analysis for the global burden of diseases, injuries, and risk factors. J Dent Res.

[2] Sheiham A (2006). Dental caries affects body weight, growth and quality of life in pre-school children. Br Dent J.

[3] Hooley M, Skouteris H, Boganin C, Satur J, Kilpatrick N (2012). Body mass index and dental caries in children and adolescents: a systematic review of literature published 2004 to 2011. Syst Rev.

[4] Hayden C, Bowler JO, Chambers S, Freeman R, Humphris G, Richards D (2013). Obesity and dental caries in children: a systematic review and meta-analysis. Community Dent Oral Epidemiol.

[5] Li LW, Wong HM, Peng SM, McGrath CP (2015). Anthropometric measurements and dental caries in children: a systematic review of longitudinal studies. Adv Nutr.

[6] Alvarez JO (1995). Nutrition, tooth development, and dental caries. Am J Clin Nutr.

[7] Narksawat K, Tonmukayakul U, Boonthum A (2009). Association between nutritional status and dental caries in permanent dentition among primary schoolchildren aged 12–14 years, Thailand. Southeast Asian J Trop Med Public Health.

[8] Benzian H, Monse B, Heinrich-Weltzien R, Hobdell M, Mulder J, van Palenstein Helderman W (2011). Untreated severe dental decay: a neglected determinant of low body mass index in 12-year-old Filipino children. BMC Public Health.

[9] Alkarimi H, Watt RG, Pikhart H, Sheiham S, Tsakos G (2014). Dental caries and growth in school-age children. Pediatrics.

[10] Anderson HK, Drummond BK, Thomson WM (2004). Changes in aspects of children’s oral-health-related quality of life following dental treatment under general anesthesia. Int J Paediatr Dent.

[11] Stephensen CB (1999). Burden of infection on growth failure. J Nutr.

[12] Psoter WJ, Reid BC, Katz RV (2005). Malnutrition and dental caries: a review of the literature. Caries Res.

[13] Psoter W, Gebrian B, Prophete S, Reid B, Katz R (2008). Effect of early childhood malnutrition on tooth eruption in Haitian adolescents. Community Dent Oral Epidemiol.

[14] Hong L, Ahmed A, McCunniff M, Overman P, Mathew M (2008). Obesity and dental caries in children aged 2–6 years in the United States: National health and nutrition examination survey 1999–2002. J Public Health Dent.

[15] Ismail AI, Sohn W, Lim S, Willem JM (2009). Predictors of dental caries progression in primary teeth. J Dent Res.

[16] Gerdin EW, Angbratt M, Aronsson K, Eriksson E, Johansson I (2008). Dental caries and body mass index by socio-economic status in Swedish children. Community Dent Oral Epidemiol.

[17] Sheiham A, Watt RG (2000). The common risk factor approach: a rational basis for promoting oral health. Community Dent Oral Epidemiol.

[18] Monse B, Benzian H, Araojo J, Holmgren C, van Palenstein Helderman W, Naliponguit EC, Heinrich-Weltzien R (2015). A silent public health crisis: untreated caries and dental infections among 6- and 12-year-old children in the Philippine National Oral Health Survey 2006. Asia Pac J Public Health.

[19] Duangthip Duangporn, Gao Sherry Shiqian, Lo Edward Chin Man, Chu Chun Hung (2016). Early childhood caries among 5- to 6-year-old children in Southeast Asia. International Dental Journal.

[20] World Health Organization (2017). The double burden of malnutrition. Policy brief.

[21] Duijster D, Monse B, Dimaisip-Nabuab JM, Djuharnoko P, Heinrich-Weltzien R, Hobdell MH, Kromeyer-Hauschild K, Kunthearith Y, Mijares-Majini MCC, Siegmund N, Soukhanouvong P, Benzian H (2017). ‘Fit for school’ – a school-based water, sanitation and hygiene programme to improve child health: results from a longitudinal study in Cambodia, Indonesia and Lao PDR. BMC Public Health.

[22] Monse B, Naliponguit E, Belizario V, Benzian H, van Palenstein Helderman W (2011). Essential health care package for children – the ‘fit for school’ program in the Philippines. Int Dent J.

[23] Monse B, Benzian H, Naliponguit E, Belizario V, Schratz A, van Palenstein Helderman W. The fit for school health outcome study: a longitudinal survey to assess health impacts of an integrated school health programme in the Philippines. BMC Public Health. 2013;13(256) 10.1186/1471-2458-13-256 PMC3610233 23517517

[24] World Health Organization (1997). Oral health surveys basic methods.

[25] Monse B, Heinrich-Weltzien R, Benzian H, Holmgren C, van Palenstein Helderman W (2010). PUFA – an index of clinical consequences of untreated dental caries. Community Dent Oral Epidemiol.

[26] Cogill B (2003). 2003 revised edition anthropometric indicators measurement guide.

[27] de Onis M, Onyango AW, Borghi E, Siyam A, Nishida C, Siekmann J (2007). Development of a growth reference for school-aged children and adolescents. Bull World Health Organ.

[28] World Health Organization [http://www.who.int/growthref/who2007_bmi_for_age/en/.] Accessed 4 July 2016.

[29] Howe LD, Galobardes B, Matijasevich A, Gordon D, Johnston D, Onwujekwe O (2012). Measuring socio-economic position for epidemiological studies in low- and middle-income countries: a methods of measurement in epidemiology paper. Int J Epidemiol.

[30] Acs G, Lodolini G, Kaminsky S, Cisneros GJ (1992). Effect of nursing caries on body weight in a pediatric population. Pediatr Dent.

[31] Cameron FL, Weaver LT, Wright CM, Welbury RR (2006). Dietary and social characteristics of children with severe tooth decay. Scott Med J.

[32] Sheller B, Churchill SS, Williams BJ, Davidson B (2009). Body mass index of children with severe early childhood caries. Pediatr Dent.

[33] Delgado-Angulo EK, Hobdell MH, Bernabe E (2013). Childhood stunting and caries increment in permanent teeth: a three and a half year longitudinal study in Peru. Int J Paediatr Dent.

[34] Duijster D, Sheiham A, Hobdell MH, Itchon G, Monse B (2013). Associations between oral health-related impacts and rate of weight gain after extraction of pulpally involved teeth in underweight Filipino children. BMC Public Health.

[35] van Cauter E, Plat L (1996). Physiology of growth hormone secretion during sleep. J Pediatr.

[36] Means RT (2003). Recent developments in the anemia of chronic disease. Curr Hematol Rep.

[37] Schroth RJ, Levi J, Kliewer E, Sellers EA, Friel J, Kliewer E, Moffatt ME (2013). Vitamin D status of children with severe early childhood caries: a case-control study. BMC Pediatr.

[38] Bansal K, Goyal M, Dhingra R (2016). Association of severe early childhood caries with iron deficiency anemia. J Indian Soc Pedod Prev Dent.

[39] Acs G, Shulmann R, Ng MW, Chussid S (1999). The effect of dental rehabilitation on the body weight of children with early childhood caries. Pediatr Dent.

[40] Monse B, Duijster D, Sheiham A, Grijalva-Eternod CS, van Palenstein Helderman W, Hobdell MH (2012). The effects of extraction of pulpally involved primary teeth on weight, height and BMI in underweight Filipino children. A cluster randomized clinical trial. BMC Public Health.

[41] Alkarimi HA, Watt RG, Pikhart H, Jawadi AH, Sheiham A, Tsakos G (2012). Impact of treating dental caries on schoolchildren’s anthropometric, dental, satisfaction and appetite outcomes: a randomized controlled trial. BMC Public Health.

[42] Heinrich-Weltzien R, Zorn C, Monse B, Kromeyer-Hauschild K (2013). Relationship between malnutrition and the number of permanent teeth in Filipino 10- to 13-year olds. Biomed Res Int.

[43] Must A, Phillips SM, Tybor DJ, Lividini K, Hayes C (2012). The association between childhood obesity and tooth eruption. Obesity Silver Spring.

[44] Alvarez JO, Navia JM (1989). Nutritional status, tooth eruption and dental caries: a review. Am J Clin Nutr.

[45] Centers for Disease Control and Prevention (2010). Use of World Health Organization and CDC Growth Charts for Children Aged 0–59 Months in the United States. MMWR.

[46] World Bank. [https://data.worldbank.org/indicator/SE.PRM.NENR.] Accessed 22 June 2018.

[47] Lipoeto NI, Geok Lin K, Angeles-Agdeppa I (2013). Food consumption patterns and nutrition transition in South-East Asia. Public Health Nutr.

[48] UNDP (2016). Support to the implementation of the sustainable development goals. United Nations Development Programme.

[49] Reeve E, Thow AM, Bell C, Engelhardt K, Gamolo-Naliponguit EC, Go JJ, Sacks G (2018). Implementation lessons for school food policies and marketing restrictions in the Philippines: a qualitative policy analysis. Glob Health.

